# A healthy settings approach to addressing painogenic environments: New perspectives from health promotion

**DOI:** 10.3389/fpain.2022.1000170

**Published:** 2022-09-27

**Authors:** Mark I. Johnson, James Woodall

**Affiliations:** ^1^ Centre for Pain Research, School of Health, Leeds Beckett University, Leeds, United Kingdom; ^2^ Centre for Health Promotion Research, School of Health, Leeds Beckett University, Leeds, United Kingdom

**Keywords:** pain, pain management, painogenic environment, health promotion, social structure, healthy-settings approach, socio-ecological

## Abstract

Structural approaches to promoting health focus on policies and practices affecting health at the community level and concentrate on systems and forces of society, including distribution of power, that foster disadvantage and diminish health and well-being. In this paper we advocate consideration of structural approaches to explore macro level influences on the burden of persistent pain on society. We argue that health promotion is an appropriate discipline to ameliorate painogenic environments and that a “settings approach” offers a crucial vehicle to do this. We encourage consideration of socio-ecological frameworks to explore factors affecting human development at individual, interpersonal, organizational, societal, and environmental levels because persistent pain is multifaceted and complex and unlikely to be understood from a single level of analysis. We acknowledge criticisms that the structural approach may appear unachievable due to its heavy reliance on inter-sectoral collaboration. We argue that a settings approach may offer solutions because it straddles “practical” and cross-sectorial forces impacting on the health of people. A healthy settings approach invests in social systems where health is not the primary remit and utilises synergistic action between settings to promote greater health gains. We offer the example of obesogenic environments being a useful concept to develop strategies to tackle childhood obesity in school-settings, community-settings, shops, and sports clubs; and that this settings approach has been more effective than one organisation tackling the issue in isolation. We argue that a settings approach should prove useful for understanding painogenic environments and tackling the burden of persistent pain.

## Introduction

Persistent pain is defined as experiencing pain for at least 3 months or beyond the normal time for tissue healing (1). The global prevalence of persistent pain is high, with estimates of one in five adults experiencing pain most days for at least 3 months (2). Previously, Johnson has discussed the notion of “painogenic environments” by exploring how an evolutionary mismatch between modern-day Anthropocene lifestyles and Palaeolithic physiological heritage may contribute to persistent pain in society (3). Indeed, one decade ago Johnson and Dixey revealed an absence of discourse between the disciplines of pain and health promotion (4). Since then, there seems to have been limited debate and discussion about the role of health promotion in addressing the burden of persistent pain in society. The reasons for this are perhaps twofold – first, the reliance of pharmacology to address painful symptoms in individuals; and second, the limited application of health promotion beyond traditional realms of addressing “lifestyle” changes.

Critics have consistently argued that health promotion, as a concept and as a practice, has been applied liberally to a range of health conditions with limited debate or consideration (5). Indeed, many have argued that applying health promotion with casual abandon is de-valuing the specific contribution it can make to improving the health and social circumstances of the most vulnerable in society (6). Those who de-subscribe from health promotion being about “lifestyle” and addressing manifestations rather than causes of the social determinants of health, argue clearly that health promotion is about individuals and communities taking greater control over their circumstances (7). While this seems utopian, many, including Marmot's body of scholarship (8, 9), have fundamentally challenged the status quo advocating for structural change to improve health (10). The notion of obesogenic environments, one which follows an ecological model of health promotion (11, 12), has caught the attention of a range of stakeholders. It is perhaps timely to re-ignite and galvanize debate on the role of health promotion in tackling other issues that could benefit from a whole-systems or structural approach.

This paper seeks to advocate consideration of structural approaches to tackle the burden of persistent pain in society by shifting away from looking at individuals, to broader “macro” influences. We suggest that health promotion may be an appropriate discipline to ameliorate painogenic environments and that a “settings approach” offers a crucial vehicle to do this. In sociology, structure refers to components or “structures” that comprise the way society, and people within society, are organised and interact, including: social class, gender, ethnicity, politics, and culture (5). Structural approaches to promoting health focus on policies and practices affecting health at the community level, with the purpose of transforming structures to improve health experience and health outcomes for people. In other words, structural approaches put a spotlight on systems and forces of society, including distribution of power, that foster disadvantage and diminish health and well-being.

## The Burden of Persistent Pain

The burden of persistent pain on society continues to rise despite major advances in medicine. Yong et al., estimated that 50.2 million adults (20.5%) in the USA reported experiencing pain on most days or every day (13). An analysis of the National Health Survey Data in the USA found that the percentage of adults with persistent pain increased from 16.4% in large central metropolitan areas to 28.1% in rural areas (14). A meta-analysis estimated that the point prevalence of persistent pain in the U.K. adult population to be 43.5% (95% confidence intervals (CIs) 38.4% to 48.6%), with moderate-severely disabling pain ranging from 10.4% to 14.3% (15). The Global Burden of Disease (GBD) project provides evidence that pain associated with musculoskeletal conditions is common, with persistent low back pain being the primary source of disability worldwide (16–18), although the precision of inferences drawn from GBD studies have been criticised because estimates were based on modelling rather than primary data (19). Nevertheless, the economic costs associated with medical and healthcare expenditures and loss of work productivity due to persistent pain is high, and has a severe impact on society (20–24).

As noted earlier, pain and health promotion do not seem to be a coherent marriage. Biomedical approaches utilising surgical, pharmacological, and non-pharmacological treatments continue to dominate clinical practice despite having potential for harmful consequences on individuals and communities through illogical prescription of drugs, including long-term opioid use, and unnecessary and inappropriate surgery (25–27). The association between persistent pain and social determinants of health, including socioeconomic status, education, occupational status, social connections etc. is undisputable (28) and recognised by professional and governmental bodies (29–31). It is widely acknowledged that optimal management of pain is *via* a biopsychosocial approach with emphasis on holistic patient-centred care with pain education and “healthy lifestyle” advice (32). In practice however, participation in and adherence to “healthy lifestyles” (such as exercise and physical activity and healthy diets) falls short of recommended levels in people with and without persistent pain, mostly because societal structures inhibit or discourage healthy behaviours (33–36).

Indeed, we argue that modern-day socio-ecological environments may hinder achievement of healthy lifestyle advice including exercise and diet because of an evolutionary mismatch between modern structures and inherited Paleolithic physiology. In other words, modern environments are “painogenic” in nature (3). This means that practitioners and decision-makers need to “zoom out” exclusively from individual approaches and perhaps consider wider impacts that determine pain.

## Painogenic Environments

In 2019, Johnson defined painogenic environments as “the sum of influences that the surroundings, opportunities or conditions of life have on promoting persistent pain in individuals or populations” (3). *Painogenicity,* the tendency to promote or contribute to (persistent) pain, acknowledges the influences that surroundings, conditions of life and/or opportunities have on the lived experience of pain of individuals in society. The idea of painogenicity and painogenic environments aligns with Boyd Swinburn's seminal work on obesogenicity, the tendency of (obesogenic) environments to promote or contribute to obesity (37). We suggest that persistent pain and obesity have similarities. Both conditions are influenced by a broad spectrum of biopsychosocial factors and managed, with only partial success, by multidisciplinary teams using biopsychosocial approaches including medical, educational, and behavioural interventions.

Living in modern society offers potential for health improvement through technological advances and digital advancements; however modern society also increases exposure to a multitude of health determinants (physical and biopsychosocial) with potential to augment the frequency, severity, quality, bodily location, and persistence of pain. These health determinants have potential to mediate, directly or indirectly, a variety of psychophysiological mechanisms with the potential to facilitate pro-inflammatory states, peripheral and central sensitisation, descending and ascending modulatory physiological systems, neuroimmune compromise, and maladaptive psychological appraisals and behavioural outcomes. Social context has a major influence on the lived experience of pain and this is acknowledged in key messages in public awareness campaigns - “*Everything matters when it comes to pain*” (https://www.flippinpain.co.uk). There has as yet, been no formal attempt to map “everything”, perhaps because of the complexity of the challenge, or because of a myopic view that solutions to the burden of persistent pain lie solely within the domain of biomedicine (38).

To date, investigation has focussed on generating domain specific knowledge about physiological (predominantly nociceptive) processes influencing the body in pain at a micro (organism) level. Far less attention has been given to generating domain specific knowledge at the macro level i.e., the influence of social, community, economic, political, cultural, and built (biosphere) environments. The coupled interaction of the macro-and micro level factors on the lived experience of pain is largely unexplored. Ultimately, socio-ecological factors are realised as changes in physiological processes (e.g. bioplasticity) and in the sense of agency driving behavioural response.

Socio-ecological conditions influence a person's lifestyle and may result in unhealthy behaviour such as sedentary routines, diets high in the ratio of omega-6: omega-3 polyunsaturated fats, carbohydrates, salt, and additives, and excessive use of recreational drugs and prescription medication. However, the situation is complex. Socio-ecological factors may augment or abate pain. For example, systematic review evidence suggests that the severity of persistent pain associated with osteoarthritis shows a positive relationship with fat and sugar intake, possibly due to pro-inflammatory mechanisms (39), yet obese people with osteoarthritis report momentary pain relief and elevated mood from eating foods high in fat or sugar, despite this being counterproductive to pain-severity in the longer term (40).

Exposure to the socio-ecological conditions of modern living is known to instigate neuroendocrine “stress” responses, and allostatic overload can result if the cumulative burden of these environmental challenges exceeds an individual's ability to cope (41, 42). A systematic review of 267 studies indicate that allostatic load and overload are associated with poorer health outcomes (43). Ramsay and Woods argue that homeostatic systems are not adapted to handle certain aspects of modern living, and the cumulative burden of chronic stress and life events leads to dysregulation of psychophysiological responses and adverse health outcomes (44). Dysregulation of the nociceptive system is known to contribute to pain that persists beyond the normal time of healing leading to significant emotional distress or functional disability, i.e. pain as a disease entity in its own right. Such chronic primary pain, which includes fibromyalgia and nonspecific low-back pain, has been included, for the first time, in the International Classification of Diseases (ICD-11); socioeconomic, cultural and ethnic influences are acknowledged as being key factors influencing symptoms (45, 46).

There is strong evidence that cumulative exposure to stressful life events in childhood is associated with poorer health outcomes and increases the likelihood of experiencing persistent pain in children and adults (47–50). Adversity during childhood generates allostatic overload that has detrimental consequences to maturing neurological, immune and endocrine systems (51) contributing to overactive stress responses, pain sensitisation, pro-inflammatory states and persistent pain in adulthood (52–54).

**Figure 1:**
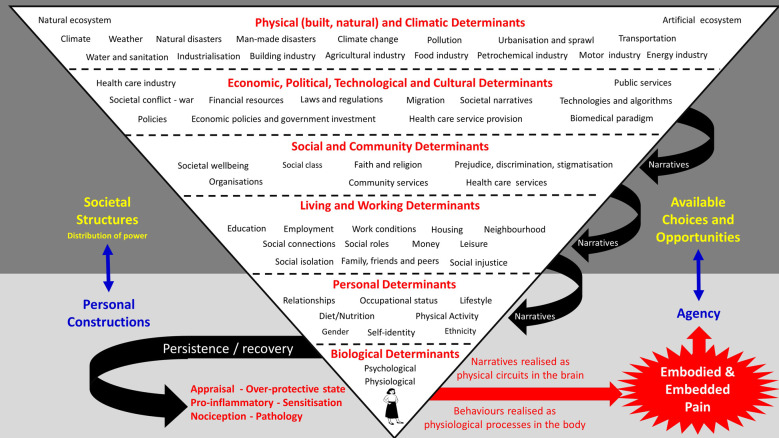
A “first step” to identify generic socio-ecological factors with potential to influence the lived experience of persistent pain and limit choices and opportunities for recovery. The schematic was developed by adapting the model of ecological development by Bronfenbrenner (55). Items (determinants) were organised within and between levels *ad hoc;* hence, the purpose of the schematic is to encourage a systematic and comprehensive analysis in the future. Attention is drawn to the role of information (words and symbols) that arise in meta-levels to form frames, metaphors, memes, and narratives, that lubricate the painogenic milieu and shape a person’s bodily self, including identity, by altering circuitry and processing in the brain (56).

Thus, we advocate using a socio-ecological lens to shed light on painogenicity and reveal macro forces impacting individuals and communities. As a first step, we identify a sample of items with painogenic potential as viewed through a broader socio-ecological framework (Figure 1).

Unpicking the influence of the complex bio-psycho-socio-ecological milieu on a person's experience of persistent pain appears overwhelming; this may be one of many reasons why attempts to tackle the burden of persistent pain remains embedded within an individual-centred biomedical paradigm. The notion of “lifestyle drift” summarises this in many ways (57) as discussed later in the paper. We believe that mapping socio-ecological factors “into the body” may offer insights to their influence of physiological processes contributing to pain. Examples include:

• industrialisation producing toxic particulates in the atmosphere that contribute to neuroimmune compromise, pro-inflammatory states, and peripheral and central sensitisation.
• Urbanisation and suburban sprawl creating reliance on motor vehicles and sedentary lifestyles resulting in painful comorbidities including pro-inflammatory states and sensitisation, and difficulties in adhering to health care professional advice to undertake more exercise.
• Economic policies contributing to socioeconomic inequalities that preclude accessibility of specialist pain management services, and a worsening pain condition.

Mapping is also likely to offer novel solutions and strategies for alleviating associated suffering and disability.

The biopsychosocial model of pain was proposed over 40 years ago, and it has proved to be a resilient construct and acknowledged within health care as the foundation of our understanding of pain and its management. Yet, treatment for persistent pain remains unimodal and embedded within a biomedical paradigm. Recently, Nicholas has called for a reappraisal of the situation (32). Exploring pain through a painogenic lens, forces attention on the role of physical, political, and sociocultural environments of modern living. To achieve this we advocate a whole systems health promotion approach in the spirit of Nettleton and Bunton's (10, *p*.44) structural critique that accounts for the physical and political environments that impact on the social environment in which the person lives. Given its strong socio-political fundamentals, health promotion may be an appropriate discipline to offer opportunities and solutions to the burden of pain.

## The role of health promotion

### The structural approach

The notion that environmental influences directly impact on the health choices that individuals make is well-understood (5). While health promotion is a broad and a contested discipline, there has been consensus from those politically drawn to the left-of-centre to see the endeavour of health promotion as being about a systems or structural change. This comprises of macro-level or environmental interventions which draws its focus towards the social, economic, political, institutional, cultural, legislative, industrial and physical environments of societies in order to modify behaviour change (58). Nettleton and Bunton (10, *p*.44) summarise:

“Essentially the structural critique argues that attempts to prevent illness and to promote health have failed to take into account the material disadvantages of people’s lives. This works at three levels: the political environment, the social environment and the physical environment.”

The structural approach avoids focusing on the individual and instead intervenes at a political or systems level to achieve positive health outcomes (59). It has potential to achieve big change in health outcomes but requires cultural and political shifts (as has been seen in smoking acceptance and cultural norms). Governments, therefore, act as stewards to create policy frameworks which encourage individuals to make healthier choices. There has been contemporary traction for this viewpoint, operationalised, for example, through the notion that “obesogenic environments” are creating adverse health outcomes for society and need to be addressed through whole-system approaches (60). Taxation has been a common way to place barriers on the purchasing of certain “unhealthy” products. The soda tax, a piece of public policy originating in the USA, was an illustration of state intervention in modifying people's consumption of sugar. Despite soda companies opposing the policy to raise taxes on sugary drinks to reduce consumption, many jurisdictions across the USA implemented this tax increase to prevent the consumption of sugary drinks and, indeed, saw reductions in consumption (61). It has been interesting to observe how “obesogenic environments” have caught the imagination of health promotion researchers, practitioners, and policy-makers. We see no strong reason why “painogenic environments” could not do the same.

The rhetoric that addressing environmental determinants of health – such as the environment; living conditions; and transport infrastructure – is well-rehearsed and yet, in countries such as the United States, the UK and Australia, there has still been a dominant view held in practice that health promotion is about modifying and addressing individual behaviour. The frequent frustration from some sections of the health promotion community is that health promotion activities are merely a “sticking plaster” for deep underlying societal problems that manifest behavioural choices (62).

#### Lifestyle drift

Several theoretical insights offer explanatory frameworks for why this occurs. The issue of “lifestyle drift” has prohibited the translation of ecological health promotion strategy to actual delivery. Lifestyle drift is the inclination for policy that recognises the need to act on upstream social determinants only to drift downstream to focus on individual lifestyle factors (63). The reasons underpinning why lifestyle drift has occurred has not been fully explored, although practical factors may be an issue. For example, lifestyle interventions are easier to devise than “upstream” interventions (57) and, moreover, lifestyle interventions are significantly easier to evaluate (64). This is certainly the case in pain practice where health promoting advice and intervention in guidelines for care remains individual-centred.

According to Green et al. (65), one of the definitive features of health promotion has been an emphasis on the environmental determinants of health (structures), but, as mentioned, this is often reduced to focus on individual choices and behaviour. The recognition, however, that the major influences on the health of an individual are outside of their immediate control has resulted in a drive to create supportive environments that are concordant with our evolutionary heritage so that the “healthy choice” is the “easy choice” (66, 67). Several international declarations on health promotion have emphasised the structural factors on people's health – the Shanghai declaration on health promotion (68) strongly emphasises the role of structural forces on health over and above the role of individual decision-making and choice.

## Implications for researchers, practitioners, policy makers and funders

### Socio-ecological frameworks

We advocate greater attention given to adapting socio-ecological frameworks, such as the Bronfenbrenner social-ecological model of human development (55), to facilitate a comprehensive approach to explore factors affecting human development at individual, interpersonal, organizational, societal, and environmental levels. The ecological orientation has grown in recent times because there has been acknowledgement that many health challenges are too multifaceted and complex to be understood from a single level of analysis (69). The approach suggests that multifaceted interventions that integrate environmental and behavioural components and that cover multiple settings and levels of analysis, are more likely to be effective in promoting personal health and public health than those narrower in scope (70).

Adapting socio-ecological frameworks to issues arising from persistent pain can identify what to address at each level. Recently, Wu et al applied the socio-ecological framework to the opioid epidemic to inform chronic pain management and successful opioid tapering for individuals living with persistent pain (71). The model of Wu et al. revealed actions for providers that could improve care of patients including recognising individual and interpersonal factors, influencing organizational policies, and shaping legal and societal issues. Wu et al. found that health care professionals are trained to assess the legitimacy of patient complaints and often consider non–life-threatening such as pain and distressing symptoms of opioid tapering of less importance. This is detrimental to a person's well-being. Wu et al. concluded that transformation in how we care for patients is needed and proposed that the focus of practitioners should be to compassionately support people living with persistent pain by empowering them in their own healing and helping them build resilience.

### Challenges when addressing structural level forces

The structural approach can be criticised to be utopian and perhaps unachievable given that it relies heavily on inter-sectoral collaboration – perhaps through town planners, health experts, decision-makers, and community groups – but it is the radical paradigm shift that may be necessary to move the challenge of persistent pain and its management away from the narrow focus on individuals. The promise of health promotion informed by socio-ecological frameworks is countered by an apparent disempowerment of health care professionals faced with the challenge of implementing structural solutions in practice. Quite simply, where would someone start? This perhaps underscores Frohlich and Potvin's criticisms that ecological models ultimately revert back to targeting individual behaviour modification (72). Similarly, Ziglio et al. note that despite the acceptance of this model, most health promotion activity has reverted to dealing with specific issues or has ignored wider social determinants (73). They suggest that the rhetoric, therefore, has failed to be a reality.

### The settings approach as a solution?

The credible critique of addressing structural level forces that impact on health is that it becomes almost impossible, or at least markedly challenging, for practitioners to address macro forces. A settings approach offers a crucial vehicle to do this and can straddle both “practical” and cross-sectorial forces that impact on people's health. Settings-based approaches to health promotion, grounded in the World Health Organization's (WHO) Ottawa Charter and Health for All strategy (7), utilises a holistic and multi-disciplinary “whole-systems approach” based on community participation, partnership, empowerment and equity [WHO - https://www.who.int/teams/health-promotion/enhanced-wellbeing/healthy-settings]. Settings-based approaches have become increasingly popular because they sit between the interface of tackling *big* structural issues (often outside of the remit of many practitioners) but in a way that is manageable and not overwhelming.

Governments have used a systems approach to develop and deliver policies to address structural level forces. For example, the Welsh Government used a systems approach to raise awareness of the detrimental impact of childhood adversity on health to target structural factors to support parents and protect children from harm. They introduced training of public service workers (e.g. teachers, police and youth officers), promoted community-led programmes to reduce adverse childhood events and improve resilience, and developed a “Support Hub” (74).

Settings-based approaches in communities have been particularly successful when supported fully at governmental levels. Sure Start, for example, was a UK Government initiative that sought to reduce and alleviate child poverty and improve health outcomes in children under 4 years and their families who live in socially deprived communities in England. Sure Start did not have a prescribed model or intervention, but it does include outreach or home visiting; family support; support for good quality play, learning, and childcare experiences; primary and community health care; advice about child and family health and development; and support for people with special needs, including help in accessing specialised services. Community participation is central to the mission of these programmes (75).

While this, of course, is not reflective of the *true* notion of an ecological model, it is an opportunity for wider synergy across social milieu. The key idea of the settings approach, or healthy settings approach, is that investments in health are made in social systems where health is not their primary remit (76). Through synergistic action between settings, it is argued that there is potential for greater health gains – including, in this case, reduced prevalence of persistent pain. Shifting back to obesity, the approach is, theoretically, relatively straightforward: childhood obesity is more effectively addressed when a range of settings work synergistically – when the school-setting, community-setting, shops and sports clubs work together to tackle the issue. This approach seems intuitively more effective than one organisation tackling the issue in isolation (77). The same *has* to be the case for the prevention and management of persistent pain.

In 2010, Australia was the first country to develop a national level holistic framework to coordinate interdisciplinary and individualised assessment, treatment, and management of acute, chronic and cancer pain (78). Subsequently, in May 2018, the Australian Government published a National Strategic Action Plan for Pain Management that endorsed a “sociopsychobiomedical prism” to view pain; the overarching goal was to minimise the pain burden for individuals and the community, and to improve the quality of life for people living with pain (79). The plan consisted of eight goals and 27 objectives. At its core was raising community awareness and knowledge about pain and its management through education to empower consumers, carers, and the wider community. The plan emphasised the need for government to recognise pain as a national and public health priority by linking pain to chronic disease frameworks in key national health and economic strategies and policies. These were to be delivered *via* “whole-of-community” engagement, and with partnerships between health care services, not-for profit organisations, researchers, the private sector, individuals, and communities.

## Conclusion

This paper has drawn on the discipline of health promotion to offer new perspectives on the conceptualisation and management of persistent pain. Compared to biomedicine, health promotion is in its infancy, but it views the experience and management of health in a more holistic way and argues that environmental factors – or structures – are as potent in their contribution to health and indeed illness than individual behaviours and choices. The application of health promotion to pain and painogenic environments has been discussed and this potentially offers future directions for the pain field. The paper suggests that socio-ecological models that address social and physical determinants of health (i.e. modern physical, social and political environments) alongside individual behaviours and practices is a sensible way to reconfigure current approaches to reducing the burden of persistent pain in individuals and communities. This will mean a move away from “health services” toward looking at other “settings” that people interact with on a regular basis. The settings-approach to health promotion is proposed here as one practical way of addressing socio-ecological factors in practical and tangible ways for practitioners and policy-makers.

Further research is needed in this field to take forward and empirically “test” or explore these ideas. Hancock (80) suggested that the settings approach is one of the most successful strategies in health promotion, but one major drawback is a paucity of high quality evaluation leading to “*an uneven and under-developed evidence base”* (81, *p*.335). St Leger (82, p100) reiterated this and argued that the approach has been legitimised more through “*an act of faith”* rather than through robust research and evaluation. If there is to be a fuller understanding of an individual's lived experience of pain in the complex environment of the modern world, we argue a need for a critical-mass of researchers working across traditional disciplinary boundaries in the future. Research areas to explore may include methodological innovation to capture how socioecological determinants impact on the exacerbation and alleviation of pain, and to ascertain lay views on how pain and societal factors impact on experiences and overall control. Such an approach to research, which relies on community-based, participatory approaches, is exceptionally common in health promotion research (83) and could be highly complementary to the pain field.

## Manuscript contribution to the field

This paper draws on the discipline of health promotion to offer new and broader perspectives on the conceptualisation and management of persistent pain. We explore how health promotion research views the experience and management of health in a more holistic way and argue that environmental factors – or structures – are likely as potent in their contribution to persistent pain as individual behaviours and choices. We discuss the application of health promotion to pain and painogenic environments to offer future directions for research in the pain field. The paper suggests that socio-ecological models that address social and physical determinants of health alongside individual behaviours and practices could reconfigure current approaches away from “health services” toward other “settings” that people interact with on a regular basis. The settings-approach to health promotion is proposed here as one practical way of addressing socio-ecological approaches in practical and tangible ways for practitioners and policy-makers. We argue that a critical-mass of researchers working across traditional disciplinary boundaries is needed in the future if there is to be a fuller understanding of an individual's lived experience of pain in the complex environment of the modern world.

## Data Availability

The original contributions presented in the study are included in the article/Supplementary Material, further inquiries can be directed to the corresponding author/s.
